# Can probiotic gargles reduce post-tonsillectomy morbidity in adult patients? A pilot, triple-blind, randomised, controlled trial and feasibility study

**DOI:** 10.1017/S0022215122000743

**Published:** 2023-03

**Authors:** M V Nasserallah, N M de Silva, V Tobin, W M Rozen, D J Hunter-Smith

**Affiliations:** 1 Department of ENT Surgery, Frankston Hospital, Peninsula Health, Frankston, Australia; 2 Department of Surgery, Frankston Hospital, Peninsula Health, Frankston, Australia; 3 Department of Plastic and Reconstructive Surgery, Frankston Hospital, Peninsula Health, Frankston, Australia; 4 Academic Unit, Central Clinical School, Monash University, Frankston, Melbourne, Australia

**Keywords:** Tonsillectomy, Postoperative Complications, Hemorrhage, Analgesia, Pilot Projects, Feasibility Studies, Randomized Controlled Trial, Double-Blind Method, Probiotics, Microbiota

## Abstract

**Objective:**

This study aimed to determine the efficacy of probiotic gargles compared with placebo gargles on reducing post-tonsillectomy morbidity in adults.

**Method:**

This was a triple-blind, randomised, controlled trial and feasibility study. Thirty adults underwent elective tonsillectomy and were randomly assigned to receive either probiotic or placebo gargles for 14 days after surgery. Daily pain scores and requirement of analgesia were measured for 14 days post-operatively. Secondary outcomes assessed probiotic safety and tolerability and the feasibility of the trial.

**Results:**

The probiotic group experienced less pain at rest on day 2. However, the amount of oxycodone (5 mg) tablets used was greater in the probiotic group compared with placebo. There were no statistically significant differences in the frequency of adverse effects between both groups. This trial was feasible.

**Conclusion:**

This pilot study suggested that probiotic gargles do not reduce post-tonsillectomy pain or bleeding, highlighting the importance of pilot and feasibility studies in clinical research.

## Introduction

Over 30 000 tonsillectomies are performed annually in Australia, making it one of the most commonly performed procedures in otolaryngology head and neck surgery.^1,2^ Despite advances in surgical techniques and peri-operative management, post-tonsillectomy morbidity continues to burden patients and the health system.^3,4^ Pain and secondary haemorrhage (bleeding occurring more than 24 hours after surgery) are the most common and significant causes of post-tonsillectomy morbidity.^5^ Post-operative pain is commonly experienced by all patients and can persist for several weeks. Post-tonsillectomy pain often results in time off work, an inability to resume normal diet and a requirement of opiate analgesics. Furthermore, the incidence of secondary haemorrhage varies from 2–40 per cent^6^ and may result in additional morbidity in the form of readmission, blood transfusion and return to the operating theatre for haemostasis.^3^

**Fig. 1.**
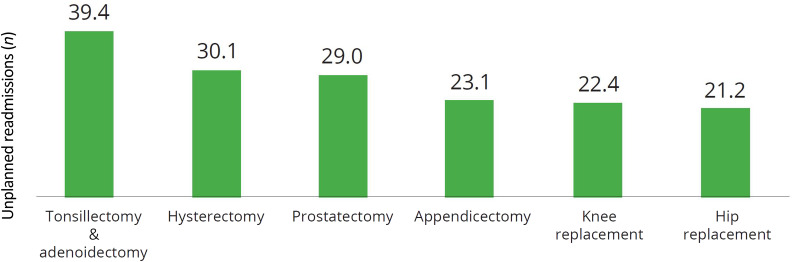
Unplanned readmissions per 1000 hospitalisations for selected procedures (2018–19). Taken with permission from Australian Institute of Health and Welfare 2020.^1^

Post-tonsillectomy morbidity is a significant health issue worldwide. Countries such as Sweden, the UK and the USA have noted steadily increasing rates of unplanned readmission following surgery.^7–9^ Unplanned or unexpected readmissions after surgery are an important metric to assess the quality and efficiency of admitted patient care in public hospitals.^1^ In Australia, tonsillectomy and adenoidectomy surgery is the most common procedure leading to unplanned readmissions (see Figure 1).^1^ In 2018 to 2019, 40 out of every 1000 tonsillectomy and adenoidectomy procedures performed in public hospitals were followed by unplanned readmission within 28 days.^1^ This rate has significantly increased since 2007 to 2008 when the rate was 26 per 1000 operations.^10^ In addition, the annual cost of unplanned hospital readmissions is estimated to be approximately A$1.5 billion nationally.^11^ Thus, the resultant economic and social costs of post-tonsillectomy morbidity are substantial. Furthermore, adults experience significantly more post-tonsillectomy morbidity than paediatric patients.^9,12^ Therefore, interventions to reduce revisits for acute pain and haemorrhage in adults should be explored.

Tonsillectomy produces an open wound that heals by secondary intention.^13,14^ This process increases the risk of developing pain and secondary haemorrhage. There are various theories behind the aetiology of post-operative pain in tonsillectomies; however, it appears to be multifactorial. Nerve irritation, inflammation and pharyngeal muscle spasm have been proposed as the underlying mechanisms responsible for this complication of tonsillectomy.^13,14^ Therefore, anything that elicits any of these three factors will worsen pain caused by tonsillectomy. Furthermore, secondary haemorrhage is caused by retraction and sloughing of the primary eschar tissue covering the healing tonsillar bed with the concomitant formation of new blood vessels. This usually occurs between day 5 and 14.^3,14^ Many factors influence post-tonsillectomy pain and haemorrhage,^6,14,15^ and the available literature examining these factors is extensive.

Although the aetiology of post-tonsillectomy pain and bleeding appears to be multifactorial, post-operative infection has been proposed as a significant contributing factor to post-operative pain and bleeding.^14,16,17^ In the first 24–48 hours after surgery, the colonisation and subsequent infection of the surgical site with oral commensals further compounds the inflammatory response and may worsen pain and bleeding risk.^14^ However, antibiotics have not demonstrated a consistent, clinically significant effect in reducing the incidence of post-operative pain or bleeding.^3^

Numerous studies have explored various surgical and non-surgical factors that influence post-tonsillectomy morbidity. However, the use of probiotic gargles to reduce post-tonsillectomy morbidity is a novel idea. Probiotics are live micro-organisms that, when administered in adequate amounts, confer health benefits on the host (International Scientific Association for Probiotics and Prebiotics in conjunction with the Food and Agriculture Organization of the United Nations and the World Health Organization).^18^ The role of beneficial bacteria on human health evolved from the work of Nobel prize laureate Elie Metchnikoff in 1908, in which he investigated the longevity and general health of a population of peasants residing in Bulgaria who consumed primarily fermented dairy products.^19^ Metchnikoff introduced the idea that lactic acid bacteria in yoghurt may counteract harmful gut pathogens and be the reason for this population's increased life-expectancy.^19^

It is now understood that the human body accommodates a very diverse and abundant collection of micro-organisms.^20,21^ These micro-organisms outweigh the number of human cells by a ratio of 10:1.^22^ Additionally, there is considerable diversity in the microbiota between body sites (e.g. oral cavity, gastrointestinal tract, skin and vagina), with each performing specific functions beneficial to the host.^23^ In 2001, Nobel prize laureate Joshua Lederberg coined the term ‘microbiome’, referring to the ecosystem of symbiotic, commensal and pathogenic microorganisms that reside in the human body.^24^ The human microbiome is composed of all the genes isolated from microorganisms (bacteria, viruses and fungi) that reside on or within human tissues and biofluids.^24^ The total number of genes in the microbiome is approximately 200-fold that of the human genome.^22^ Therefore, it is clear that symbiosis with these micro-organisms seems to be a condition for survival.^24^

The human microbiome is influenced by a host of endogenous and exogenous factors. These include genetics, diet, antibiotics, smoking, alcohol consumption, socioeconomic status and pregnancy.^20^ Any disruption in the human microbiome, also known as dysbiosis, may alter the normal function of that organ and result in disease.^22^ Therefore, a logical management approach to situations that alter our microbiota would be to deliberately increase our association with specific non-pathogenic organisms to maintain microbial homeostasis.

Our understanding of the structure and function of the human microbiome in both diseased and healthy states has significantly improved with the development of new gene-sequencing techniques. These innovative genomic technologies, such as shotgun metagenomics and next-generation sequencing techniques, have vastly revolutionised the throughput and accuracy of DNA sequencing of the microbiome in human samples compared with conventional culture-based detection methods.^20,25^ As a result, the body of scientific literature to support the use of probiotics to maintain human health and prevent disease has exponentially risen over the past 20 years.^21^

The *Streptococcus salivarius* K12 probiotic strain has demonstrated an ability to reduce the growth of pathogenic organisms commonly implicated in upper respiratory tract infections.^26,27^ Furthermore, *in vitro* and *in vivo* studies have also demonstrated that the K12 strain possesses immunomodulatory and anti-inflammatory properties that can upregulate the human body's innate immune response and concomitantly suppress the release of pro-inflammatory cytokines.^28,29^ Therefore, the use of a probiotic strain that is native to the oral cavity, such as *S salivarius* K12, may reduce post-tonsillectomy morbidity.

This pilot and feasibility study was the first trial exploring the role of probiotics in reducing post-tonsillectomy morbidity. The efficacy of a 14-day course of probiotic (*S salivarius* K12) gargles in reducing adult post-tonsillectomy morbidity was compared with placebo gargles. The hypothesis was that this was a feasible trial and that probiotic gargles were safe, tolerable and would reduce post-tonsillectomy pain and bleeding. Finally, an ancillary analysis comparing gargles versus no gargles (using data from a similar study^30^) was conducted to explore whether the act of gargling itself can reduce post-tonsillectomy pain.

## Materials and methods

### Study design

This study was a pilot, prospective, triple-blind, randomised, controlled trial and feasibility study. The trial was performed at a single-centre (Frankston Hospital, Australia) between October 2019 and February 2021 in an adult population (aged 18–55 years), with a parallel group study design using an allocation ratio of 1:1.

**Fig. 2.**
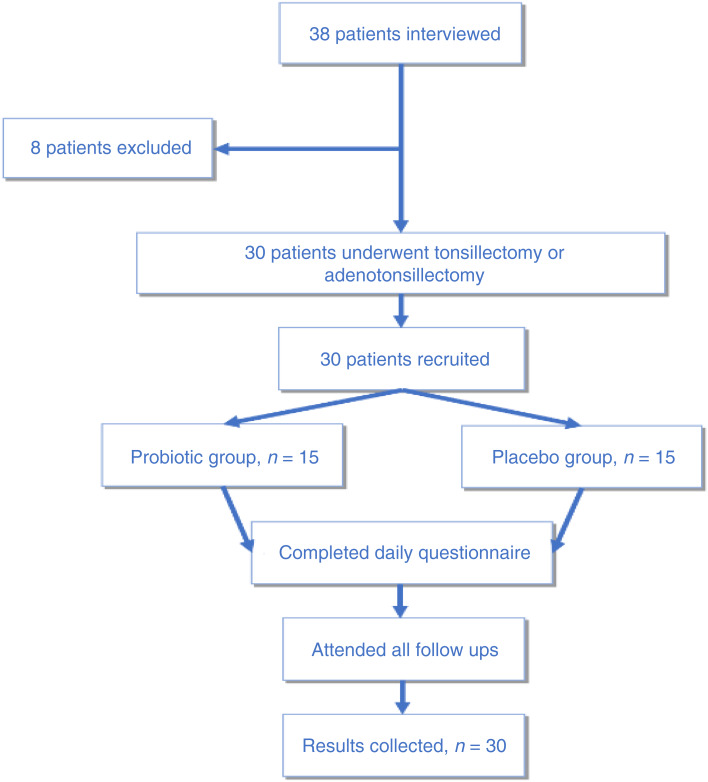
Consolidated Standards of Reporting Trials Flow diagram. Thirty patients were recruited and divided into each arm, with no dropouts.

All procedures contributing to this work complied with the ethical standards of the Australian guidelines. This study was approved by the human research ethics committee of Peninsula Health (protocol number: HREC/51745/PH-2019) and the Department of Health and Ageing, Therapeutic Goods Administration (trial number: CT-2019-CTN-03230-1). The trial was registered with the Australian New Zealand Clinical Trials Registry (trial identification number: ACTRN12619001474145) and was performed according to the Consolidated Standards of Reporting Trials 2010 extension to randomised pilot and feasibility trials. The investigation complied with the Helsinki Declaration of 1975, as revised in 2008. Screening, recruitment and study retention for this study is summarised in a consort diagram (Figure 2).

### Eligibility and selection of participants

All participants were adult patients scheduled for elective tonsillectomy or adenotonsillectomy on the ENT surgical waiting list at Frankston Hospital. Pre-operatively, 38 patients were interviewed in the out-patient clinic by the operating surgeon from October 2019 to February 2021.

Patients were selected only if they satisfied the inclusion criteria. Patients included were aged 18–55 years, weighed more than 50 kg, and were scheduled to undergo either tonsillectomy or adenotonsillectomy, with indications for surgery being tonsillar hypertrophy, asymmetrical tonsillar enlargement, recurrent tonsillitis or previous quinsy.

The exclusion criteria for this trial included: allergies to non-steroidal anti-inflammatory drugs, opioids, codeine, oxycodone or paracetamol; lactose intolerance; unstable metabolic diseases or disorder; history of endocarditis; immunocompromised patients; kidney, liver or cardiovascular disease; haemorrhagic diathesis; requirement for other concomitant surgical procedures; recent use of probiotics within one month; patients taking regular non-steroidal anti-inflammatory drugs, paracetamol or opiates; and women who were pregnant, nursing or trying to conceive. Eight patients were excluded as they did not satisfy these criteria.

### Informed consent

Informed consent was obtained by the participant signing the patient information and consent form at the pre-operative visit before surgery.

### Operative method

Thirty participants were recruited and randomly assigned to each study arm (15 patients per group). A single experienced surgeon performed all operations under general anaesthesia with the use of orotracheal intubation. Tonsillectomy with or without adenoidectomy was performed with the use of the Boyle–Davis mouth gag. The post-nasal space was inspected and adenoidectomy was performed, if clinically necessary, using curettage or a suction diathermy device at 25 W (using a Valleylab Force FX^™^ Electrosurgical Generator C). Tonsils were then removed using electrocautery, with monopolar diathermy set at 15 W for dissection and bipolar cautery set at 12 W for haemostasis. On induction of general anaesthesia, all patients received a single dose of 1.2 g of intravenous (IV) benzylpenicillin (or 600 mg of IV clindamycin if allergic) to reduce the risk of bacteraemia and eliminate the presence of pathogenic organisms.^31^ Furthermore, antibiotic pre-treatment is used to eliminate pathogenic pathogens, allowing live therapeutic bacteria to colonise the target site better when using probiotics.^32^ Peri-operative parecoxib and dexamethasone were used for all patients to reduce pain and improve recovery in the immediate post-operative period.^33^

### Peri-operative care

After surgery, patients were transferred to the post-anaesthesia care unit for monitoring and then moved to the ward once they were awoken and deemed safe. During the post-operative period, patients received regular analgesics, with allowance for oral oxycodone for breakthrough pain relief, ondansetron and metoclopramide as antiemetics and other medications as deemed necessary.

### Randomisation and blinding

Prior to the study's commencement, the trial pharmacist generated a randomised order that dictated the allocation of the trial intervention (probiotic powder) and placebo (isomalt powder) to the recruited participants in chronological order. This order was generated using the randomisation function in Microsoft Excel® spreadsheet software. Block randomisation was used with a predetermined ratio of 1:1 for the 30 participants. Once a patient was identified as eligible to participate in the trial by the study investigator, the patient's details were provided to the trial pharmacist. Each trial participant was then given a trial pack (which contained the probiotic or placebo) and was allocated a unique identification number (1 to 30) by the pharmacist packaging the medication. The pharmacist was the only person who had information on what product each patient was receiving until the completion of data collection.

Patients were allocated to either probiotic gargles or placebo gargle groups according to the generated randomisation schedule. The probiotic BLIS K12 Daily Defence Junior powder (containing 125 million colony forming units per scoop of *S salivarius* K12; BLIS Technologies, Dunedin, New Zealand) and placebo powder (isomalt) were identical in appearance and taste. They were pre-packed in identical bottles and consecutively labelled with a unique identification number for each participant according to the randomisation schedule. The allocation sequence and bottle contents were concealed from the investigator enrolling and assessing participants.

This information was kept in sealed envelopes, stored in a locked cupboard in the hospital pharmacy. The investigators, participants and statistician were all blinded to the allocation process.

### Post-operative care and instructions

After morning breakfast, all participants were discharged home with regular medications and the trial intervention (probiotic gargles or placebo gargles). Study medications were distributed by an independent pharmacist at the hospital with no clinical involvement in the study. Patients were advised to dissolve two pre-measured scoops of the trial powder into 20 ml of warm water, gargle for 30 seconds and then swallow. Participants were instructed to gargle 4 times a day for 14 days, ideally 30 minutes after brushing teeth and before meals. Regularly prescribed medications consisted of 1 g of paracetamol (4 times a day for 10–14 days), 5–10 mg of oxycodone (4-hourly as required for rescue analgesia), 10 mg of metoclopramide (3 times a day as needed for nausea and vomiting) and aperients as needed. Participants were also advised to consume regular rough textured foods, coordinate analgesia consumption approximately 30–60 minutes before meals and avoid strenuous activity two weeks after surgery.

### Daily questionnaires and follow up

Participants were sent an electronic survey (daily 14-day questionnaire) to their mobile or e-mail via SurveyMonkey® daily (see Appendix 1). This questionnaire was specifically designed for this trial but was adapted from a similar study using a paper form.^30^ Alternatively, a paper form of the diary (see Appendix 2) was provided to the participant if they did not have access to mobile or e-mail. Only one participant preferred completing the paper form of the diary.

Patients were followed up on days 5, 14 and 28 post-operatively. Day 14 and day 28 out-patient visits were conducted in person at Frankston hospital, and patients were followed up via telephone on day 5. On day 14, participants returned any unused powder from the trial intervention, which was delivered to and weighed by the trial pharmacist to measure compliance. If a participant required additional information or analgesia, they were advised to speak to the study investigator at any time from day 1 to day 28 after surgery.

### Coronavirus disease 2019 precautions

As a result of coronavirus disease 2019 (Covid-19) restrictions on trial continuity and trial protocols, the following amendments were made. (1) Day 5 follow-up was via telephone as per protocol. (2) Day 14 and day 28 follow ups were in the out-patient clinic as per protocol. (3) Before the out-patient visit, the associate investigator contacted patients and asked if they had experienced any fever or respiratory symptoms, were close contacts with Covid-19 suspected or proven patients or if they had tested positive for Covid-19. (4) If patients had no risk factors, they were seen in the out-patient clinic as planned. The associate investigator wore gloves and mask and eye protection during the consultation and conducted appropriate hand hygiene. The patient was also advised to wear a mask and conduct appropriate hand hygiene. (5) At the end of the consultation, a disinfectant wipe was used to wipe down all surfaces contacted by the patient and the investigator, and appropriate hand hygiene was completed. (6) If the patient had any of the above risk factors, the associate investigator advised them to have a Covid-19 polymerase chain reaction swab test if they had not had one already and to remain self-isolated until the result was negative before reviewing them in the out-patient clinic. If this was not feasible, a telephone consultation was completed using the same questions from the day 5 telephone consultation. (7) If participants could not attend the out-patient clinic, they were advised to bring back the remaining powder to the pharmacy on day 14 as per protocol. If participants could not return unused medication themselves, a family member was asked to do so. Powder containers were handled with gloves and appropriate hand hygiene when returning to the trial pharmacist.

### Outcomes measures

#### Primary outcome

The primary outcomes assessed were post-tonsillectomy pain and the requirement of opiate analgesia. Participants recorded average daily pain scores at rest and for drinking and eating over the preceding 24-hour period, with a numerical rating scale (0 being no pain to 10 being the worst pain experienced) for 14 days after surgery. Additionally, the daily requirement of paracetamol and opiate analgesics was recorded as the number of 500 mg tablets of paracetamol and 5 mg tablets of oxycodone (Endone®) that the participant consumed daily for 14 days after surgery. These outcomes were recorded using the ‘daily 14-day questionnaire’.

#### Secondary outcome

The secondary outcomes assessed the safety and tolerability of probiotics. The readmission rates because of pain or dehydration were recorded using hospital records. The rate of post-tonsillectomy haemorrhage was assessed using the ‘daily 14-day questionnaire’ and by examination of the participants’ hospital records. A four-point haemorrhage scale was used to categorise the severity of bleeding (1 = no bleeding; 2 = minimal bleeding less than a mouthful, managed at home with ice gargles; 3 = moderate bleeding, managed medically in hospital with gargle or silver nitrate cautery without blood transfusion requirement; 4 = profuse bleeding, requiring the operating theatre or needing a blood transfusion). The number of side effects experienced daily (e.g. nausea, vomiting, constipation, drowsiness, halitosis, abdominal pain) was also documented by participants in their ‘daily 14-day questionnaire’.

### Feasibility measures

#### Design

The feasibility of the study's design was determined by examining the following factors: the ability and time required of study staff to coordinate recruitment, screening and clinic tasks; the ability of staff to contact participants; the duration of the initial phone call to participants; and the number of staff required for the initial recruitment phone call. Screening and clinic tasks were also assessed by recording the number of staff required and the duration (in minutes) of interviews at participant visits on day 5 (via phone), day 14 and day 28 (in the out-patient clinic).

#### Recruitment and screening

The ability to recruit and screen participants was assessed by measuring the time and people required to be screened to enrol 30 participants to completion of the final trial visit.

#### Randomisation

The balance of characteristics in each group determined the trial's ability to perform successful randomisation.

#### Adherence

Adherence in both groups was assessed by: the amount of probiotic or placebo gargle consumed as measured by participant diaries and bottle return, their attendance at follow-up appointments and their completion of daily questionnaires.

#### Safety

Safety was assessed by the number and description of serious adverse events (any admission to the emergency department or hospitalisation, life-threatening events or results in significant morbidity or death) and other adverse events. Serious adverse events (including haemorrhage requiring operative intervention or blood transfusion, readmission because of pain and dehydration or septicaemia) were adjudicated by an independent medical monitoring team (two independent ENT specialists) as either unrelated, possibly related, probably related or definitely related to trial intervention.

#### Retention

The study design was assessed for number of participants that withdrew in each treatment arm.

### Ancillary analysis

An exploratory analysis comparing the effect of placebo gargles versus placebo tablets on pain scores and the requirement of analgesics post-tonsillectomy was also performed. A co-author of our study (NMdS) provided anecdotal evidence that probiotic gargles significantly reduced post-tonsillectomy pain. Therefore, this additional analysis was performed to delineate whether the act of gargling can reduce post-operative pain compared with no gargles. The raw data for participants who consumed placebo tablets from a recently published study was obtained with the author's permission.^30^ This study was a double-blind, placebo-controlled, randomised, controlled trial conducted in the same setting (Peninsula Health) and utilised a similar study design as our trial. This earlier study explored the efficacy of celecoxib in reducing post-tonsillectomy pain compared with placebo. Pain scores were assessed using a numerical rating scale (0–10) and the requirement of oxycodone in both the amount of 5 mg tablets consumed and the number of days. Oxycodone was used in the placebo gargle and placebo tablet groups. However, as these outcomes were reported for only up to 10 days post-operatively in the celecoxib study, these outcomes from both groups (placebo gargles and placebo tablets) were assessed and compared for 10 days after surgery.

### Statistical analysis

The statistician remained blinded to trial intervention allocation while performing the statistical analysis. Once data collection was complete, the pharmacist was notified to provide a list of the two separate treatment groups by their identification numbers without revealing which group was the intervention and the placebo. The excel spreadsheet data was de-identified by deleting each participant's name and unit record number and was henceforth identified only by the study identification number. As mentioned previously, the statistician performed the analysis on two groups of patients, without the knowledge of which group was allocated to the probiotic and which group was allocated to the placebo.

The collected data was entered into a Microsoft Excel® spreadsheet software before being imported into Stata (version 16, StataCorp, College Station, USA) for statistical analysis. The distribution of continuous data was determined using the Shapiro–Wilk test for normality. Normal data has been presented as mean ± standard deviation (SD) and non-normal as median (interquartile range). Comparisons of normally distributed continuous variables were made using *t*-tests or repeated measure analysis of variance (ANOVA) with Bonferroni post hoc tests. Continuous data that failed the Shapiro–Wilk test for normality were compared using the Wilcoxon Mann–Whitney or Kruskal–Wallis tests. Categorical or interval data have been presented as percentage frequency and assessed using Fisher's exact test. *P*-values less than 0.05 were assessed as statistically significant.

## Results

### Demographic data

From October 2019 to January 2021, 30 adult patients (18–55 years old) were enrolled in the study, with 15 in the probiotic group and 15 in the placebo group. There were no statistically significant differences between these treatment groups in demographic or surgical characteristics (Table 1), including age, sex, indication of surgery and surgery type (tonsillectomy *vs* adenotonsillectomy).

**Table 1.** Demographic and surgical characteristics

Parameter	Probiotic	Placebo	*P-*value
Age (median (interquartile range); years)	30 (12)	25 (5)	0.31
Male (*n* (%))	3/15 (20)	1/15 (6.7)	0.30
Indication (*n* (%))			0.21
– Recurrent tonsillitis	12/15 (80)	9/15 (60)	
– Tonsillolith	3/15 (20)	1/15 (6.7)	
– Recurrent peritonsillar abscess	0	3/15 (20)	
– Sleep-disordered breathing	0	1/15 (6.7)	
– Asymmetric tonsillar hypertrophy	0	1/15 (6.7)	
Surgery type (*n* (%))			0.43
– Tonsillectomy	9/15 (60)	12/15 (80)	
– Adenotonsillectomy	6/15 (40)	3/15 (20)	

### Pain scores

**Fig. 3.**
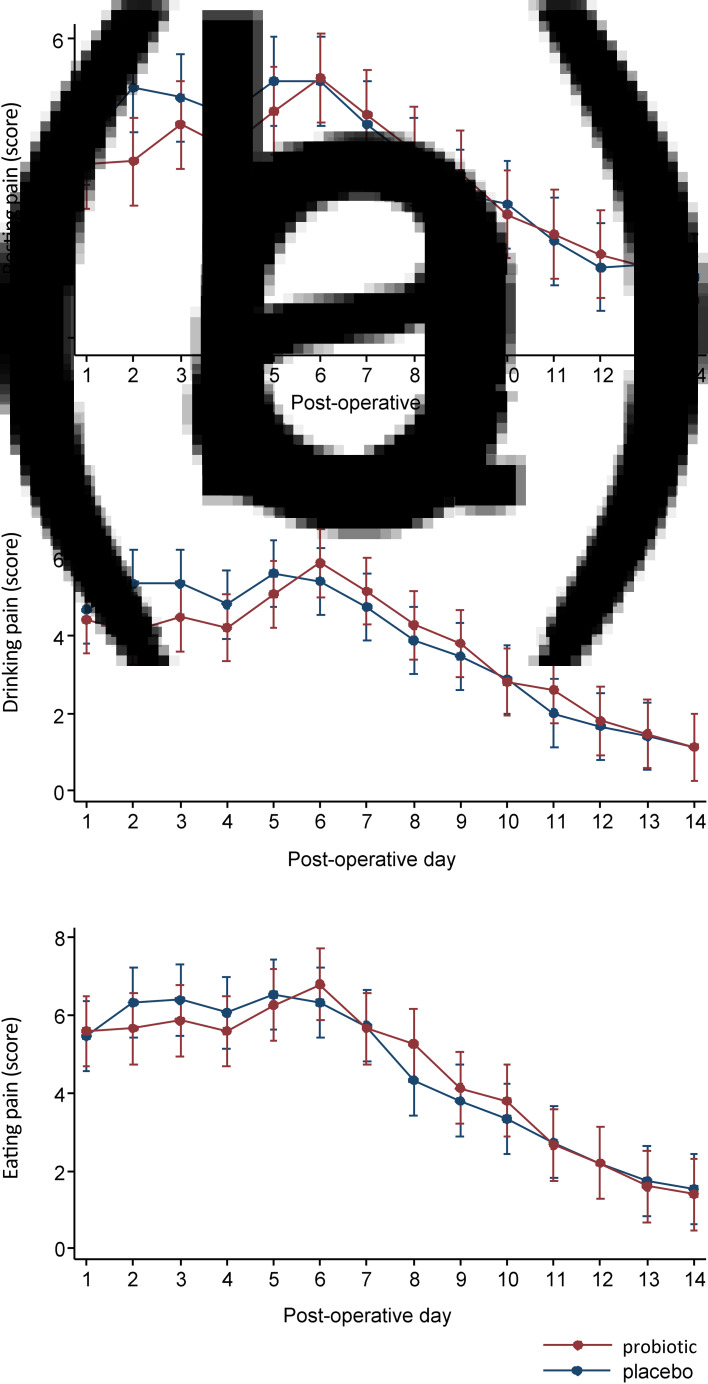
Mean pain scores at (a) rest, (b) drinking and (c) eating from day 1 to day 14. *Only the mean pain score at rest on day 2 alone was statistically significantly lower in the probiotic group compared with the placebo group (mean ± standard deviation, probiotic versus placebo group: 3.5 ± 1.8 *vs* 5 ± 2, respectively; *p* = 0.02 by repeated measures analysis of variance).

Post-operative pain scores during rest and eating and drinking were reported during the first 14 post-operative days (Figure 3). There was no statistically significant difference in pain scores at rest on each day (Figure 3a) except on day 2, with statistically significantly lower pain scores in the probiotic group (probiotic *vs* placebo, mean ± SD: 3.5 ± 1.8 *vs* 5 ± 2, respectively; *p =* 0.02 by repeated measures ANOVA). No statistically significant differences were observed in the daily pain scores while drinking (Figure 3b) or eating (Figure 3c). Although there was a trend for lower reported pain scores during the first five post-operative days at rest and for drinking and eating in the probiotic group compared with the placebo group (data pooled from days 1–5, data not shown), this difference was not significant.

### Oxycodone use

**Fig. 4.**
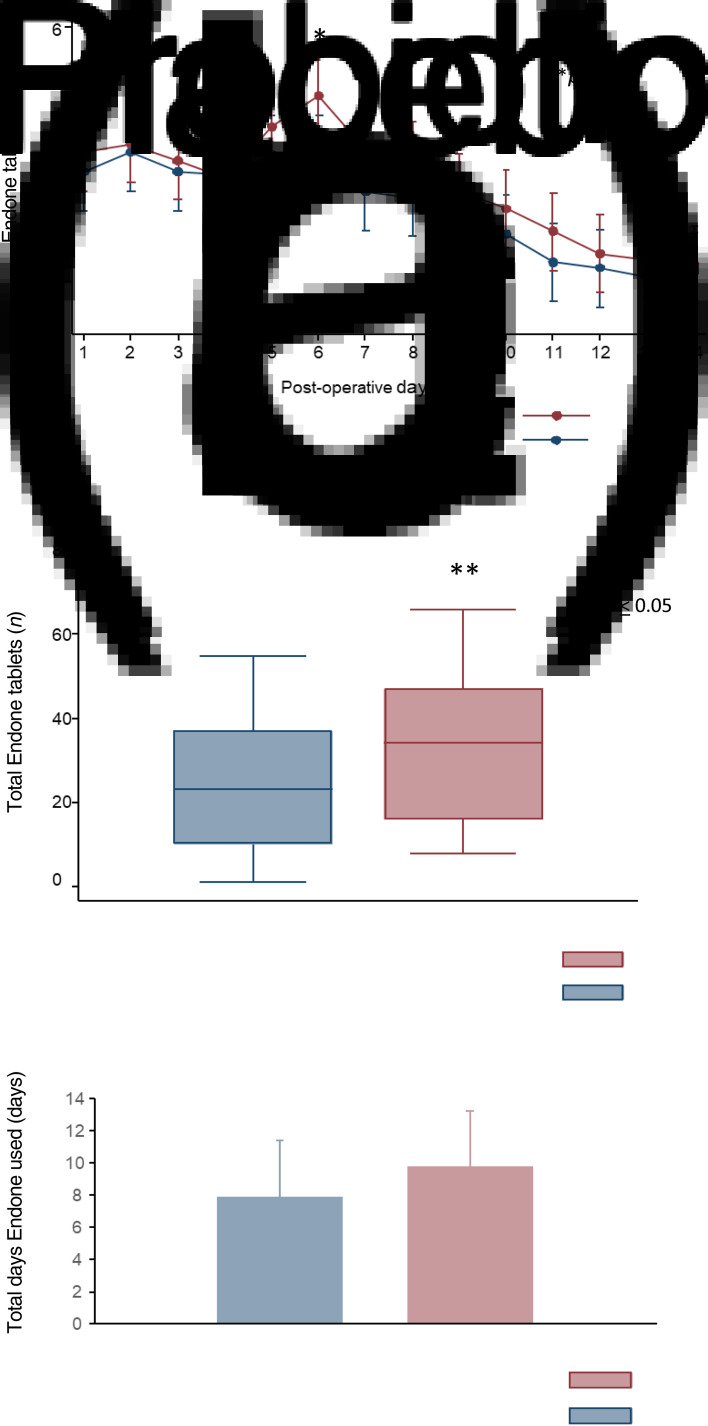
(a) Daily mean number of oxycodone (Endone®) tablets used. (b) Median total number oxycodone tablets. (c) Mean total number of days oxycodone used day 1 to 14. *Mean number of oxycodone tablets used was statistically significantly higher in the probiotic group only on day 6 (probiotic *vs* placebo, mean ± SD: 4.4 ± 2.8 *vs* 3 ± 2.3, respectively; *p =* 0.03 by repeated measures analysis of variance). **Total number of oxycodone tablets used was statistically significantly higher in the probiotic group (probiotic group *vs* placebo median and interquartile range: 34 (31) *vs* 23 (27), respectively; *p =* 0.00 by Mann–Whitney test).

The reported daily number of 5 mg oxycodone tablets (Figure 4a), total number of oxycodone tablets (Figure 4b) and the total number of days (Figure 4c) that oxycodone was used for each group over the post-operative 14 days were examined to provide an overall estimation for the requirement of analgesia.

Although the average number of oxycodone tablets taken on day 6 was higher in the probiotic group compared with the placebo group (mean ± SD for number of oxycodone tablets used for probiotic *vs* placebo (day 6): 4.4 ± 2.8 *vs* 3 ± 2.3, respectively; *p =* 0.03 by repeated measures ANOVA), there were no statistically significant differences between the treatment groups detected on any other day (Figure 4a). The median total number of oxycodone (5 mg) tablets (Figure 4b) used was statistically significantly higher in the probiotic group compared with the placebo group (total number of oxycodone tablets used (day 1 to day 14, median (interquartile range): 34 (31) *vs* 23 (27), respectively; *p =* 0.00 by Wilcoxon Mann–Whitney test). Finally, there were no statistically significant differences (*p =* 0.14) in the mean total number of days that oxycodone was used between the groups, with the probiotic group using oxycodone for 9.8 ± 3.4 days (mean ± SD) compared with 7.9 ± 3.5 days in the placebo group (Figure 4c).

### Paracetamol use

**Fig. 5.**
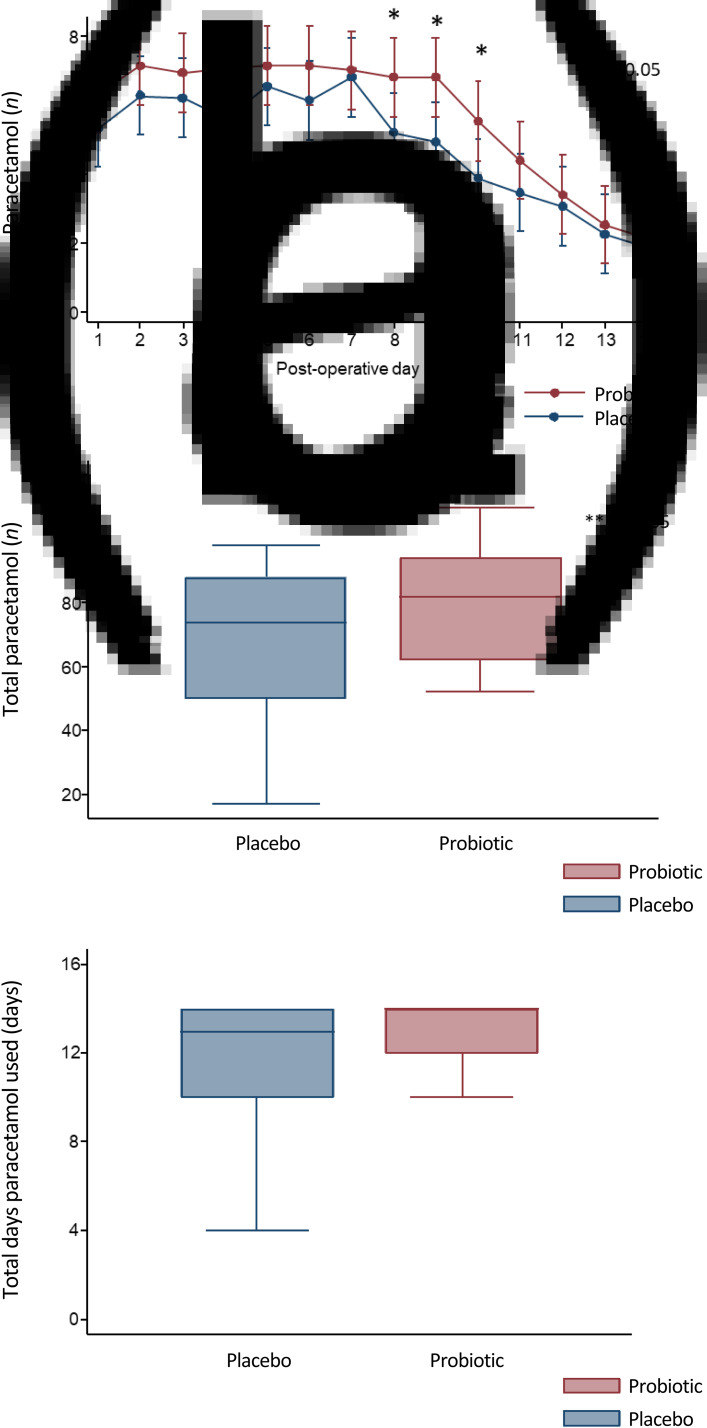
(a) Mean daily number of paracetamol tablets used, (b) median total number of paracetamol tablets used and (c) median total number of days paracetamol used. *Mean number of paracetamol used was statistically significantly higher in the probiotic group on days 8, 9 and 10 (probiotic *vs* placebo, mean ± SD: day 8: 6.8 ± 1.8 *vs* 5.2 ± 2.7, *p =* 0.05; day 9: 6.8 ± 1.7 *vs* 4.9 ± 3.2, *p =* 0.02; and day 10: 5.5 ± 2.3 *vs* 3.9 ± 3, *p =* 0.04 by repeated measures analysis of variance. **Total number of paracetamol tablets used day 1 to day 14 was statistically significantly higher in the probiotic group (median interquartile range for probiotic group *vs* placebo: 82 (32) *vs* 74 (38), respectively; *p =* 0.00 by Mann–Whitney test).

Although the daily average number of paracetamol tablets taken on days 8, 9 and 10 was statistically significantly higher in the probiotic group compared with the placebo group (amount of paracetamol used (mean ± SD) on day 8: 6.8 ± 1.8 *vs* 5.2 ± 2.7, *p =* 0.05; day 9: 6.8 ± 1.7 *vs* 4.9 ± 3.2, *p =* 0.02; and day 10: 5.5 ± 2.3 *vs* 3.9 ± 3, *p =* 0.04, respectively), there were no statistically significant differences on any other post-operative day (Figure 5a).

Both the total amount of 500 mg paracetamol tablets consumed (Figure 5b) and the total number of days paracetamol was used (Figure 5c) over the 14 days after surgery were also calculated to provide additional measures for the requirement of analgesia. The median total number of paracetamol (500 mg) tablets (Figure 5b) used was statistically significantly higher in the probiotic group compared with the placebo group (amount of total paracetamol used (day 1 to day 14), median (interquartile range): 82 (32) *vs* 74 (38), respectively; *p =* 0.00). However, there was no statistically significant difference in the median total number of days paracetamol was used between the two groups (Figure 5c).

### Side effects

**Fig. 6.**
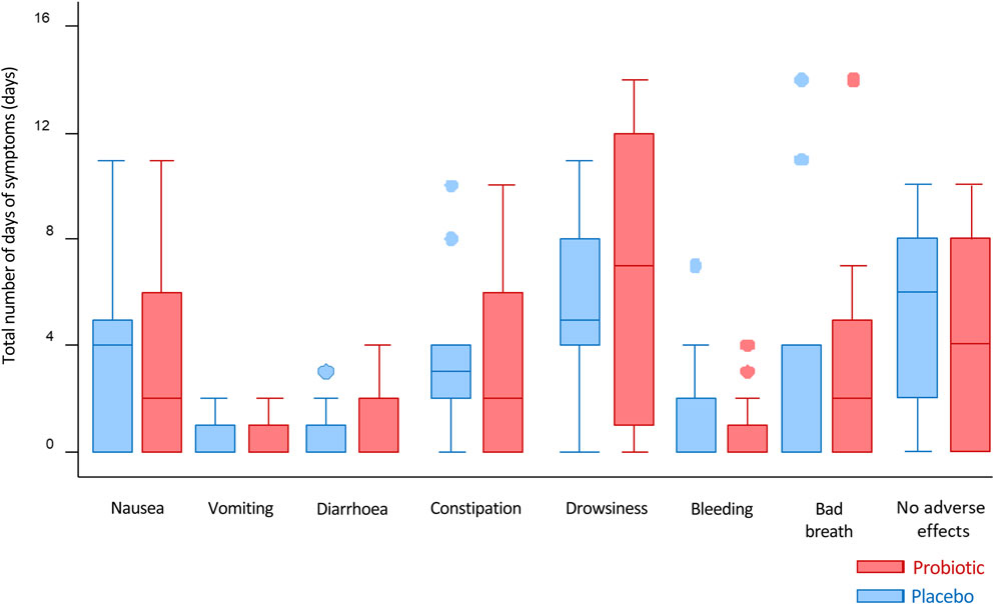
Median total number of days the various adverse effects were reported. There were no statistically significant differences in the number of days that any of the side effects were reported between both groups.

Participants recorded whether they experienced any of the following side effects daily: nausea, vomiting, diarrhoea, constipation, drowsiness, bleeding and bad breath. The total number of days of reported adverse effects are presented by group. There were no statistically significant differences in the total number of days that any of the side effects were reported between the probiotic and placebo groups (Figure 6).

### Haemorrhage rates

The incidence of post-tonsillectomy haemorrhage using a four-point haemorrhagic scale was assessed over the 28 days post-surgery. No patients had a primary haemorrhage event (occurring equal to or less than 24 hours post-surgery). Six patients (3 in each treatment group) required readmission because of secondary haemorrhage but did not require operative intervention or blood transfusions (*p =* 1.00). There were no statistically significant differences between the two groups with respect to the frequency of the haemorrhage categories (*p =* 0.48; Table 2).

**Table 2.** Post-tonsillectomy haemorrhage classification

Bleeding	Probiotic (*n* (%))	Placebo (*n* (%))	*P-*value
1 – None	10/15 (66.7)	7/15 (46.7)	0.48
2 – Minimal	2/15 (13.3)	5/15 (33.3)	
3 – Moderate	3/15 (20)	3/15 (20)	
4 – Profuse	0	0	

### Readmission for pain or dehydration

The number of participants requiring admission because of pain or dehydration was assessed over the 28 days post-surgery. Only one patient in the trial was admitted with pain, and they had received the probiotic treatment (*p =* 1.00). This patient presented on day 6 with thick tonsillar slough in bilateral tonsillar fossa and 10 out of 10 pain. The thick slough was suctioned, and the patient was given two doses of 8 mg IV dexamethasone and discharged the same day.

### Feasibility measures

#### Design

There were no changes made to the study design or study protocol for the trial duration. Minor modifications were made to the consultation protocols as a consequence of the Covid-19 pandemic.

#### Recruitment, screening and randomisation

Thirty-eight patients from the hospital waiting list were screened, of which 8 were excluded. All 30 patients were randomised to either the probiotic group or placebo group and completed the daily questionnaires and attended all mandatory follow-up appointments. The expected time to recruit 30 participants was 6 to 12 months when the trial was designed. However, because of restrictions on elective surgery as a result of the Covid-19 pandemic, study recruitment was completed in 15 months. Successful randomisation was achieved with the assistance of the Department of Pharmacy at Frankston Hospital. There were equal numbers of participants in each group with no significant differences in baseline characteristics.

#### Adherence

We had a 99 per cent attendance rate to all follow-up appointments and 100 per cent completion of daily questionnaires. Furthermore, all participants had used over 80 per cent of the trial intervention as calculated by the weight of the returned powders, and there was no statistically significant difference in the percentage of treatment powder used between the probiotic and placebo groups (Table 3).

**Table 3.** Percentage of treatment powder used by each group

	Probiotic (mean ± SD; %)	Placebo (mean ± SD; %)	*P*-value
Percentage treatment powder used	83.5 ± 11.6	88 ± 12	0.3

SD = standard deviation

#### Safety

There were four serious adverse events in the intervention group and three in the control group (six patients readmitted because of bleeding and one patient readmitted because of pain). The independent medical monitoring team (consisting of two ENT surgeons) were notified of any serious adverse event and were asked to assess the relatedness of the event to the trial intervention. The overall consensus was that the events were most likely unrelated or only possibly related to the trial intervention. No other serious adverse events were reported.

#### Retention

A participant was considered lost to follow up if they failed to return for either day 14 or day 28 scheduled visits and could not be contacted by the trial site staff. There were no patients lost to follow up during this trial.

### Ancillary analysis

**Fig. 7.**
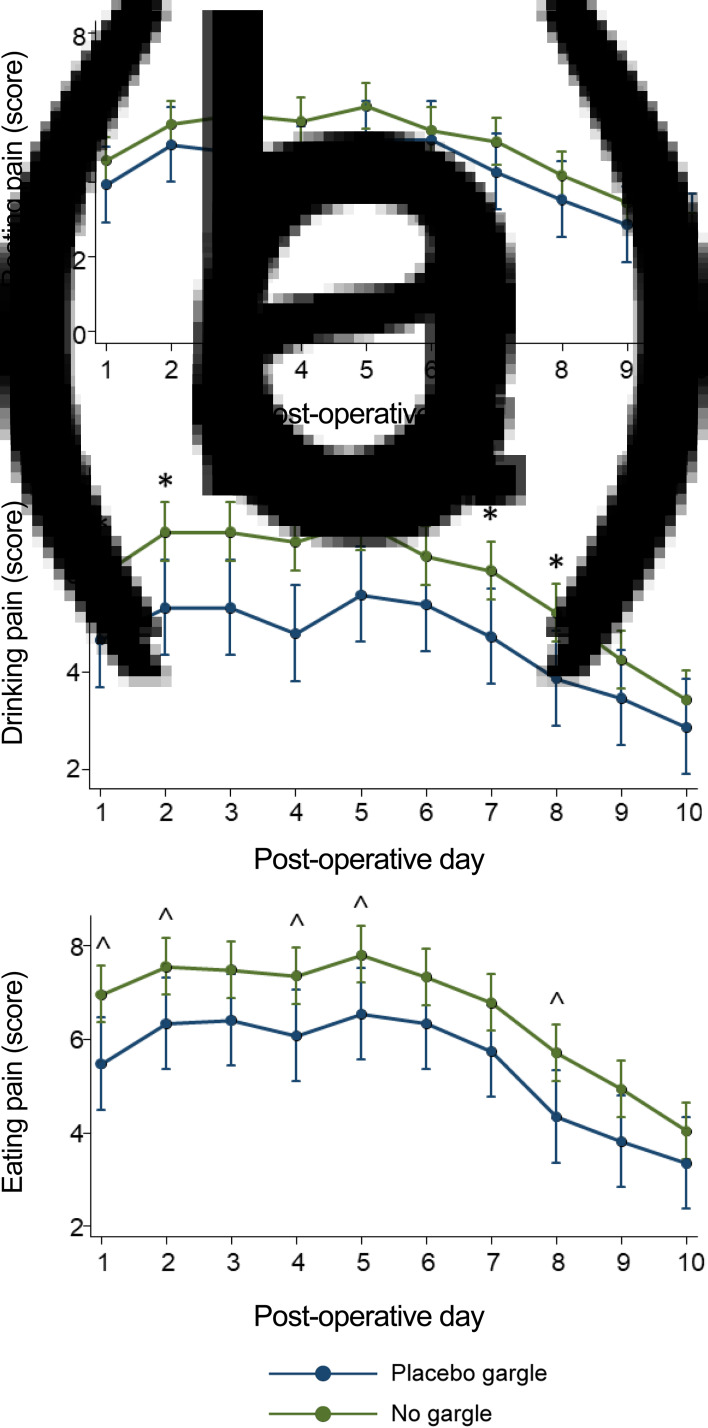
Mean pain scores at (a) rest, (b) drinking and (c) eating from day 1 to day 10. *The mean pain score while drinking was statistically significantly lower in the placebo gargle group compared with the placebo tablet group on days 1–5, 7 and 8 (*p <* 0.05 by repeated measures analysis of variance). ˄The mean pain score while eating was statistically significantly lower in the placebo gargle group compared with the placebo tablet group on days 1, 2, 4, 5 and 8 (*p <* 0.05 by repeated measures analysis of variance).

Post-operative pain scores during rest, eating and drinking were reported during the first 10 post-operative days for both placebo gargle and placebo tablet groups (Figure 7). There was no statistically significant difference in pain scores at rest (Figure 7a). However, the placebo gargle group had significantly less post-operative pain on days 1–5, 7 and 8 while drinking (Figure 7b) and on days 1, 2, 4, 5 and 8 while eating (Figure 7c) compared with the placebo tablet group. In addition, there was no significant difference in the number of oxycodone tablets used between both groups (total number of oxycodone tablets (5 mg) used (day 1 to day 10, median (interquartile range): placebo gargle = 23 (26) *vs* no gargle = 18.5 (20); *p =* 0.31). Furthermore, there were no statistically significant differences in the number of days oxycodone was used post-operatively (total number days oxycodone used, median (interquartile range): placebo gargle = 7 (4) *vs* no gargle = 9 (3); *p =* 0.62).

## Discussion

The economic and social costs associated with post-tonsillectomy morbidity are significantly increasing, as demonstrated by the rise in unplanned readmission rates following tonsillectomy and adenoidectomy surgery over the past decade.^1,7–9,34^ Therefore, interventions to offset revisits for acute pain and secondary bleeding should be explored to decrease adult tonsillectomy morbidity. This pilot and feasibility study explored the efficacy of probiotic (*S salivarius* K12) gargles in reducing post-tonsillectomy morbidity in adults. We have successfully demonstrated that this trial was feasible. However, probiotic (*S salivarius* K12) gargles did not reduce post-tonsillectomy pain, requirement of analgesia or adverse effects compared with placebo gargles.

Post-tonsillectomy morbidity is influenced by a host of dependent (e.g. age, sex, indication) and independent (e.g. operative method, co-morbidities, infection) variables. Hence, many trials have explored various peri-operative and post-operative factors that may help reduce post-tonsillectomy morbidity. Our study hypothesised that probiotic gargles would reduce post-tonsillectomy pain and bleeding by their ability to reduce surgical-site infections and decrease inflammation.

Probiotics have emerged as a fruitful area of research in both health-related and commercial targets over the past 30 years. Probiotics are defined as ‘live micro-organisms that, when administered in adequate amounts, confer a health benefit on the host’.^18^ The health benefits associated with probiotics have been mainly observed in gastrointestinal diseases.^35^ Probiotics demonstrated a reduction in post-operative morbidity in colorectal, upper gastrointestinal and liver transplantation surgery in two recent meta-analyses.^36,37^ Probiotics and synbiotics (combined prebiotics and probiotics) demonstrated reduced post-operative surgical-site infections, pneumonia, sepsis, antibiotic use, abdominal distension, diarrhoea and urinary tract infections in these studies. The ability of probiotics to modulate the gut-immune response and produce short-chain fatty acids have been proposed as the underlying mechanisms leading to the reduction in post-surgical complications.^36,37^

The role of probiotics in managing post-operative pain, however, is relatively novel. A pilot study demonstrated that a multi-strain probiotic containing *Levilactobacillus brevis* CECT7480 (KABP-052) and *Lactoplantibacillus plantarum* CECT7481 (KABP-051) significantly reduced mean pain scores compared with placebo following mandibular third molar extraction on days 5, 6 and 7 post-operatively.^38^ This analgesic effect was attributed to the ability of *L brevis* CECT7480 strain to produce high levels of γ-aminobutyric acid *in vitro*. However, the requirement of analgesia between groups was not assessed.^38^ Furthermore, probiotics can reduce inflammatory pain by inhibiting pro-inflammatory cytokines, increasing anti-inflammatory cytokines and eliciting the expression of μ- and κ-opioid receptors in peripheral nerve fibres, thereby decreasing hypersensitivity and facilitating analgesia.^39^

The evidence supporting probiotics in maintaining oral health and preventing disease is also rapidly expanding.^23^ Probiotics have demonstrated a reduction in dental caries, periodontal disease, peri-implantitis, oral candidiasis and halitosis.^23,40^ Furthermore, there is evidence that probiotics can prevent and reduce upper respiratory infections.^41^ Although researchers initially tried to establish whether conventional approved intestinal probiotics could also influence the oral microbiome, these bacteria could not inhabit the oral mucosa. Thus, any oral cavity health benefits seem transitory and attributable primarily to immune stimulation via action on the gastrointestinal tract.^42^ Given the difference in the microbial communities found in the upper respiratory tract and the gastrointestinal tract,^25^ a more logical strategy is to utilise microbes isolated from their natural oral habitat in healthy humans as oral probiotics.

*S salivarius* is one of the earliest colonisers of the oral cavity and remains a predominant commensal inhabitant in adults.^42^ In 2001, the K12 strain became the first *S salivarius* to be commercially developed as a probiotic, and more than 50 million doses have now been marketed internationally by the New Zealand company BLIS Technologies (Dunedin, New Zealand). The *S salivarius* K12 strain competitively binds to human surface epithelial cells and prevents the growth of pathogenic organisms.^42^ In addition, this strain produces two lantibiotic bacteriocins, salivaricin A2 and salivaricin B, also known as bacteriocin-like inhibitory substances.^42^ These bacteriocins can effectively counteract the growth of group A beta-haemolytic *Streptococcus pyogenes.*^26,27^ Additionally, the K12 strain can also inhibit the growth of many other potential pathogens of the upper airway that cause pharyngotonsillitis, acute otitis media and halitosis.^27,43^ Furthermore, *in vitro* and *in vivo* studies have demonstrated that the K12 strain possesses anti-inflammatory properties by its ability to inhibit the nuclear factor κB pathway, interfering with pro-inflammatory cytokine synthesis and suppressing interleukin-6, interleukin-8 and tumour necrosis factor-α secretion.^28,29^ The safety of strain K12 has been supported by a series of studies affirming the absence of known virulence factors and antibiotic resistance determinants, its low mutagenicity predisposition, acute and subacute toxicity testing in rats, and a successful high-dosage trial in humans.^40,44^

*S salivarius* K12 gargles did not cause a reduction in post-tonsillectomy pain, bleeding or other adverse events in our trial. There are several plausible explanations as to why we did not observe a significant difference between both groups.

This trial did not provide evidence that the K12 strain colonised the tonsillar fossa as neither microbiological cultures nor quantitative polymerase chain reaction analyses of saliva or the tonsilla fossa were performed during the trial. However, in previous studies, the ability of the K12 strain to colonise and persist in saliva samples was successfully demonstrated after consuming *S salivarius* K12 (BLIS Technologies) in both lozenge^45^ and dissolved powder^44^ forms. Furthermore, we advised patients to gargle and rinse their mouth for 30 seconds with the gargle product to achieve optimal results. This advice was provided by BLIS lead scientists, Professor John Tagg and Doctor John Hale. Hence, the K12 strain was assumed to have colonised the oral cavity of patients who used the probiotics gargles.

This study utilised a single species (*S salivarius* K12) probiotic instead of a multi-strain or multi-species probiotic, which may have also potentially reduced the probiotic efficacy. The Human Microbiome Project identified over 700 species of bacteria that reside as commensals in the oral cavity.^23^ As a result, several companies have developed various probiotic mixtures to create greater genetic diversity to restore the ‘natural’ microbiota. However, the comparative efficacy of mono-strain and multi-strain probiotics has not been adequately studied yet.^46^ The advantage of multi-strain probiotics comes mainly from bacterial synergistic interactions, which enhance their positive effect on the host organism.^46^ One such study demonstrated that the combination of *Lactobacillus salivarius* LS01 and *Bifidobacterium breve* BR03 led to a more pronounced immunomodulatory effect on peripheral blood mononuclear cells of asthmatic patients (*in vitro)* than if individual strains were used.^47^

Additional mutual effects of probiotic strains depend on their mutual inhibition and tolerance to each other.^46^ A seminal study by Chapman *et al.* showed significant cross inhibition of growth amongst *Lactobacillus* strains, suggesting that multiple strains used together are less effective at inhibiting pathogens, such as *Clostridium difficile* and *Escherichia coli,* than single strains.^48^ In contrast, Bifidobacterium strains have been shown to tolerate each other and be more effective at inhibiting pathogens when used in combination.^48^ Furthermore, *in vivo* studies comparing the effectiveness of mono-strain and multi-strain probiotics have also yielded contrasting results.^46^ Multi-strain probiotics demonstrated greater efficacy in preventing gastrointestinal infections in children and adults when compared with mono-strain probiotics.^46^ However, a recent systematic review highlighted that a probiotic multi-strain preparation resulted in negative outcomes in patients with irritable bowel syndrome.^49^ Other trials utilising single-strain probiotics, such as bifidobacteria, have shown positive results.^49^ Although tolerance and mutual inhibition of bacteria can be predicted by their genus affiliation,^48^ the advantages of mono-strain or multi-strain probiotics remain largely unclear. The compatibility of strains should be tested prior to producing commercial products to avoid reducing their effectiveness. In this study, we chose a single-strain probiotic as there is limited evidence on the efficacy of probiotic mixtures containing multiple streptococcal species or strains.

The optimal dose (as indicated by colony forming units of *S salivarius* K12) required to produce a clinical benefit is unknown. A dose of 250 million *S salivarius* K12 colony forming units (equivalent to 2 scoops of BLIS Daily Defence Junior powder, BLIS Technologies) four times a day was selected, which equates to a maximum total dose of 1 billion colony forming units/day. This dose has been demonstrated as safe and tolerable in adults who used a daily dose for 28 days.^44^ Additionally, the ability of probiotic strains to colonise different niches is dose-dependent, as demonstrated by Burton *et al*.^50^ This study demonstrated that the probiotic *S salivarius* M18 strain colonised the mouth significantly more in the highest dosage group (1 x 10^9^ colony forming unit/dose/day) compared with lower dosage groups. Burton *et al*. proposed that the increased ability of a probiotic to persist at the target site will likely allow the organism to have a more significant impact on the host, translating into a greater clinical benefit.^50^

The dose regimens adopted so far are based on standards for the gastrointestinal tract and paediatric healthcare. Ouwehand (2017) explored the dose-responses of probiotics in various human studies.^51^ There was a consistently significant dose-response in patients with antibiotic-associated diarrhoea, with studies reporting less incidence of antibiotic-associated diarrhoea with a higher dose.^51^ Nevertheless, an important and consistent finding in probiotic research is that relatively few organisms, compared with the number of organisms inhabiting the niche into which they enter, can confer health benefits. This observation is evident for the densely populated gastrointestinal tract (with over 10^14^ residing bacterial organisms), where a trace amount of probiotics (10^8^–10^10^ colony forming unit) is supplied.^52^ Ritchie and Romanuk (2012) conducted a meta-analysis showing that the effect of dosage on probiotic efficacy for gastrointestinal diseases was relatively minor. Furthermore, Ouwehand's (2017) review reported no clear dose-response with probiotics in the prevention or management of *Clostridium difficile* associated diarrhoea, irritable bowel syndrome, necrotising enterocolitis or atopic dermatitis. Although there was an absence of a clear dose-response for these conditions, the available evidence was of low quality, making it difficult to draw firm conclusions.^51^ Future large-scale, randomised, controlled trials are required to determine whether a dose-effect exists in the use of probiotics in various clinical scenarios.

Finally, the role of infection in the aetiology of post-tonsillectomy pain and bleeding remains unclear. The colonisation of the tonsillar fossa with commensal bacteria can result in infection and a worsening inflammatory response.^14^ Animal and human models assessing wound healing post-tonsillectomy have demonstrated that bacteria colonising the tonsillar fossa within the first 24–48 hours post-operatively further compounds the inflammatory response.^14^ However, a Cochrane review by Dhiwakar *et al.* (2012) showed there is no evidence to support a consistent, clinically significant impact of systemic antibiotics in reducing the main morbid outcomes (pain and bleeding) following tonsillectomy.^3^ Furthermore, the studies to support topical antibiotics in reducing post-tonsillectomy morbidity are inconsistent and have several biases because of methodological flaws.^53,54^ Although some studies show that antibiotics reduce the bacterial count in the post-operative tonsillar fossa,^16,55^ a clinical correlation in terms of reduction in morbidity is lacking. Hence, Dhiwakar *et al.* (2012) suggest that post-tonsillectomy complications primarily occur as a result of tissue injury and oedema induced by surgical technique, with minimal or nil additional morbidity conferred by bacterial inflammation.^3^ Therefore, the role of probiotics in managing post-tonsillectomy morbidity may also be limited.

### Strengths and limitations

We successfully demonstrated the feasibility of this study (in study design, recruitment, screening, randomisation, adherence, safety reporting and retention) and provided clear evidence that a future large-scale randomised, controlled trial is not recommended. Pilot and feasibility studies are critical because they influence the design of the main trial. Additionally, feasibility studies may avoid significant potential costs associated with conducting large-scale research, especially when pilot trials demonstrate that there may be no clinical benefit related to an intervention.

As this was a pilot and feasibility study, no prior power estimations were performed. However, we believe that selecting 30 patients has adequately demonstrated the feasibility of assessing the efficacy of probiotic gargles on reducing post-tonsillectomy morbidity. Although a statistical significance was reported for between-group differences, we acknowledge that this study's small sample size limits the validity and generalisability of our results.

Finally, there is some evidence that post-tonsillectomy haemorrhage is more common in patients with recurrent tonsillitis than in those with tonsillar hypertrophy or obstructive sleep apnoea.^56^ In this study, there was no statistically significant difference in the surgical indications between the two groups. However, the probiotic group had more patients with recurrent tonsillitis than the placebo gargle group (80 per cent *vs* 20 per cent), which could have skewed the results. Nonetheless, no high-quality studies have been conducted to determine the risk factors for post-tonsillectomy bleeding in adult patients. Several retrospective studies^6,57,58^ could not demonstrate that surgical indication was a significant risk factor for post-tonsillectomy haemorrhage in adult patients. Future research is needed to characterise the tonsil microbiome in adult patients undergoing surgery for various indications (recurrent tonsillitis, obstructive sleep apnoea, tonsilloliths) to determine whether this has an effect on post-operative outcomes.

### Recommendations to future study designs

If a large-scale study is to be conducted, we recommend recruiting a larger number of participants following a power analysis to minimise statistical error. At least one additional clinical staff member would be required to coordinate recruitment, screening and follow up. Finally, a quantitative analysis of culture swabs from the oral cavity and tonsillar fossa should be taken to assess if probiotics successfully colonise the surgical site. Future studies should also evaluate the efficacy of multi-strain versus mono-strain probiotics and the optimal dose required to produce a therapeutic benefit.

- Post-tonsillectomy morbidity is a significant health issue that continues to burden patients and the public health system
- Probiotics are live micro-organisms that, when administered in adequate amounts, confer a health benefit on the host
- *Streptococcus salivarius* K12 strain can inhibit the growth of pathogenic organisms in the upper respiratory tract and possesses anti-inflammatory and immunomodulatory properties
- *S salivarius* K12 gargles did not reduce post-tonsillectomy pain, bleeding or other adverse events compared with placebo gargles
- Gargles may help reduce post-tonsillectomy pain compared with no gargles
- Pilot and feasibility studies are invaluable tools for conducting novel research

Furthermore, our ancillary analysis demonstrated significantly reduced pain while drinking and eating in patients using placebo gargles compared with no gargles (Figure 7). Thus, we hypothesise that performing post-tonsillectomy gargles with inert substances (such as saline or betadine) may help reduce post-tonsillectomy pain, possibly by continuously debriding the surgical wound. Future studies should explore the role of gargles versus no gargles in reducing post-tonsillectomy morbidity.

## Conclusion

In this small pilot study, probiotic (*S salivarius* K12) gargles did not reduce post-tonsillectomy pain, the requirement of analgesia or adverse effects compared with placebo gargles. Furthermore, this paper highlights the importance of pilot and feasibility studies in their ability to investigate areas of uncertainty regarding novel research ideas.

The role of probiotics in health and disease is a rapidly emerging field of science and is continuously yielding exciting discoveries. Next-generation sequencing techniques have improved the throughput and accuracy of DNA sequencing of the genomes of microbial communities in human samples, allowing for the development of probiotics that can target specific health problems. Future studies exploring the role of probiotics in human disease should determine the efficacy of multi-strain products and whether a dose-dependent effect exists.

## Appendix 1. Electronic survey (daily 14-day questionnaire)

**Table:**

**Pain diary 14-day questionnaire**

In this diary you will be asked to rate your pain each day while resting, eating and drinking, record any side effects, medications used and the number of medications used. Please complete this survey after eating dinner. Please record your answers over past 24 hrs (i.e. 7:00 pm day before to 7:00 pm today).

**Question Title**

*1. Please write your full name

**Question Title**

*2. Please write your study ID number (located on your trial pack).

e.g if Study ID is #12, please write 12

**Question Title**

*3. On a scale of 0–10, please rate your pain while at rest over the past 24 hrs?

**Table:**

0	1	2	3	4	5	6	7	8	9	10
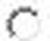

**Question Title**

*4. On a scale of 0–10, please rate your pain while drinking over the past 24 hrs?

**Table:**

0	1	2	3	4	5	6	7	8	9	10

**Question Title**

*5. On a scale of 0–10, please rate your pain while eating over past 24 hrs?

**Table:**

0	1	2	3	4	5	6	7	8	9	10

**Question Title**

*6. Please record how many tablets of Endone (5 mg) did you consume over past 24 hrs? (7:00 pm yesterday to 7:00 pm today)

e.g. 0 for none, 8 for eight

**Question Title**

*7. Please record how many tablets of Paracetamol (500 mg) did you consume over past 24hrs? (7:00 pm yesterday to 7:00 pm today)

e.g. 0 for none, 8 for eight

**Question Title**

*8. Please record how many powdered gargles you consumed over past 24 hrs? (7:00 pm yesterday to 7:00 pm today)

e.g. 0 for none, 4 for four

0

1

2

3

4

**Question Title**

*9. Did you take any other medications today (e.g. maxolon, coloxyl and senna) over the past 24hrs?

Next

**Question Title**

10. If you answered yes, please record what medication you took and how many tablets you took over past 24 hrs (7:00 pm yesterday to 7:00 pm today)?

e.g. 1 Maxolon, 2 ondansetron

**Question Title**

*11. Please select the box if you experienced any of the following side effects (more than one selection allowed)?

If you did not experience any side effects please select none.

 Vomiting

 Nausea

 Diarrhoea

 Constipation

 Bleeding

 Drowsy

 Bad breath

 None

Prev Done

## Appendix 2. Information and instructions for patients

Medications

There are 3 medications

**Probiotic/placebo**

Dissolve 2 scoops (1/2 teaspoon) of powder in 20 ml of warm water, gargle for 30 seconds then swallow.

How often?   Four times a day, 4–6 hours apart

How long for?   Fourteen (14) days

**Paracetamol**

How many?   Two tablets (1 g)

How often?   Every six (6) hours, maximum of 8 tablets a day

How long for?   Ten (10) days

You can stop taking this if there is no pain

**Endone**

How many?   One–two tablet (5 mg)

Endone is a strong pain killer. Take it only if your pain is not controlled with regular paracetamol and powdered gargle.

Take 4–6 hours apart

Maximum dose per 24 hours is four tablets (20 mg)

**The Pain Diary**

The post-operative day is the number of days after you have had your tonsillectomy. For example –

**Table:**

January 1st	Day of surgery
January 2nd	Post-operative day 1
January 3rd	Post-operative day 2
January 4th	Post-operative day 3

Please complete the diary at the end of each day (just before you go to bed):

Record all pain scores based on pain experienced during dinner. There are 3 types of pain you will need to record:

**Table:**

1	Rest	The level of pain you experience when resting
2	Drink	The level of pain you experience when drinking
3	Eat	The level of pain you experience when eating

Record the name and dose of any other medications that you are taking

Record how many times you have used the gargle that day

Record how many paracetamol tablets you took that day

Record how many endone tablets you took that day

List any side effects using the following codes

**Table:**

V	Vomiting
N	Nausea
D	Diarrhoea
C	Constipation
B	Bleeding
D	Drowsy
H	Bad breath

Make a note of any other issues that arise relating to your post-tonsillectomy period such as visits to your GP or the emergency department for bleeding

Record the day you stopped taking probiotic/placebo if it is less than 7 days post-surgery

Record the day you stopped taking paracetamol if it is less than 10 days post-surgery

Your review with your surgeon will be

Post-operative day 5–7 via phone

Post-operative day 14 in person at Frankston Hospital

Post-operative day 28 in person at Frankston Hospital

**Post Tonsillectomy Diary**                          **Date of surgery:** _________

**Table:**

Post-operative Day	Pain score during dinner (0–10)			No. of Endone taken / day	No. of paracetamol taken/day	No. of gargles used	Side effects (N/V/C/D/B)	Other medications
	Rest	Drink	Eating					
Example	5	8	8	3	8	3	N,C	1 Maxolon
1								
2								
3								
4								
5								
6								
7								
8								
9								
10								
11								
12								
13								
14								
Day & date when there is no pain on drinking					E.g.: day 7, 8/1/13			
Day & date when there is no pain on eating								
Day & date when you are pain free								
Day & date when you return to school/work								

*Should you have any queries, please call Frankston Hospital 03-9784 7777 and ask the operator to put you through to Michael Nasserallah, ENT Registrar.
